# Lipid Clinics Are Urgently Required in the Iranian Public Health System

**Published:** 2010

**Authors:** Majid Ghayour-Mobarhan, Seyyed Mohammad Reza Kazemi-Bajestani, Gordon Ferns

**Affiliations:** 1 Cardiovascular Research Center, Avicenna (Bu-Ali) Research Institute, Mashhad University of MedicalScience, Mashhad, Iran; 2 Department of Nutrition, Faculty of Medicine, MUMS, Mashhad, Iran; 3 Radboud University Nijmegen Medical Center, Nijmegen, The Netherlands; 4 Institute for Science and Technology in Medicine, University of Keele, Stoke on Trent, Staffordshire, ST4 7QB, United Kingdom

**Keywords:** Lipid Clinics, Secondary prevention, Dyslipidemia

## Abstract

**Objectives:**

Lipid Clinics are specialized centers for clinical assessment and follow up of patients with dyslipidaemia in order to deliver an acceptable improvement in their lipid profiles. We assessed the changes in lipid profile of dyslipidemic patients attending a Lipid Clinic over a 1 year period on lipid-lowering therapy.

**Methods:**

Dyslipidemic patients (n=238) were recruited from the Lipid Clinic at the Royal Surrey County Hospital, Guildford, UK. All patients were regularly seen at the clinic and the compliance of lipid-lowering drug consumption, prescribed by the consultant was assessed over a period of one year.

**Results:**

The mean age of the patients was 55.2 ± 0.86 years and the male/female ratio was 143/95. The lipid profiles of patients attending the Lipid Clinic over the period of one year of close monitoring changed significantly. Triglyceride, total cholesterol and low density lipoprotein cholesterol were reduced by 27.04%, 20.48% and 22.67%, respectively (P<0.001) and high density lipoprotein cholesterol rose by 8.96% (P<0.001); the 10-year calculated coronary risk factor of all patients decreased significantly (39.29%, P<0.001).

**Conclusions:**

Our findings confirmed the effectiveness of a Lipid Clinic in the management of lipid profile and cardiovascular risk of dyslipidemic patients.

## INTRODUCTION

The relationship between dyslipidemia and atherosclerosis is well-established.^1^,^2^,^3^ Atherosclerosis is the major cause of coronary artery disease (CAD) and stroke, the principal cause of mortality and morbidity in developed nations, and this is also likely to be the case in developing countries.^4^ Lowering low density lipoprotein cholesterol (LDL-C) has been the main intervention for cardiovascular prevention strategies. This approach has been demonstrated to be effective in both primary and secondary prevention.^5^ Prospective epidemiological studies have also shown that serum high density lipoprotein cholesterol (HDL-C) levels are a strong and independent risk factor, being inversely related to cardiovascular risk,^6^ whilst an elevated serum triglyceride concentration is a significant risk factor for CAD in both sexes.^7^ Statin therapy is a key intervention for the improvement of lipid profile and plays an important role in primary and secondary prevention of cardiovascular diseases.^8^ Several previous studies have shown that statins are relatively safe and major adverse effects are not expected following the long-term consumption of these drugs.^8^–^10^ Lipid and cardiovascular risk reduction clinics (CRRC) are an important means of providing regular follow up and expert care for patients with dyslipidaemia. The clinical effectiveness of CRRCs in normalizing the serum LDL-C levels of the dyslipidemic have been shown in previous studies in which they were compared to primary care physicians or non-lipid clinic specialists.^11^–^14^ Olsen et al. (2005) demonstrated that the patients who were under the care of CRRCs were also found to have significantly lower levels of other traditional risk factors including hypertension and hyperglycemia.^15^ To highlight the role of lipid clinics in follow-up care, we carried out this study to investigate the likely improvement in lipid profile over a period of one year monitoring of dyslipidemic patients in a university lipid clinic center in United Kingdom.

## METHODS

Dyslipidemic patients (n=238) were recruited from the Lipid Clinic at the Royal Surrey County Hospital, Guildford, UK. Patients were monitored regularly at intervals of 4-6 months over the period and compliance with their lipid lowering drugs and dietary changes were monitored. Each patient gave written informed consent to participate in the study. The study protocol was approved by the South-West Surrey Research Ethics Committee and the Advisory Committee of Surrey University.

### 

#### Blood sampling

Blood samples were collected between 08:30 and 10:30 am after a 12-h fasting by venipuncture of the antecubital vein. Samples for lipid profile were taken into plain vacutainer tubes, and those for measurement of glucose were taken into vacutainer tubes containing fluorideoxalate. All chemicals were obtained from Sigma Chemical Co (Poole, United Kingdom) unless stated otherwise.

#### Analytic methods

A fasting lipid profile, comprising total cholesterol, triacylglycerols, and HDL-C was obtained for each patient. Lipids and glucose were measured by routine methods using a Bayer Advia 1650 analyzer (Bayer, Newbury, United Kingdom). LDL-C was calculated for all subjects except the patients with serum triacylglycerol concentrations >4.0 mmol/L using the Friedewald formula.^16^

#### Statistical analysis

Statistical analysis was undertaken with the use of MINITAB software (release 13; Minitab Inc, State College, PA), with determination of descriptive statistics (i.e., means, medians, SEMs, and interquartile ranges) for all variables. Comparisons before and after treatments were assessed by paired t-test for normally distributed data, or by Mann-Whitney test for nonparametric data.

## RESULTS

The mean age of the patients was 55.2 ± 0.86 years and the male/female ratio was 143/95 (Table 1). Eighty-two patients were obese [body mass index >30 kg/m^2^]; 42 were diabetic (fasting plasma glucose concentration >7 mmol/L); 55 had established coronary artery disease (CAD), and 186 were hypertensive. Of the latter group, 76 had systolic blood pressure (SBP) ≥160 mmHg or diastolic blood pressure (DBP) ≥100 mmHg. One hundred and ten patients had SBP between 130 and 160 mmHg or DBP between 85 and 100 mmHg. One hundred and seventy-six patients were hypertriglyceridemic (serum triacylglycerol concentration >1.8 mmol/L), and 216 patients were hypercholesterolemic (serum total cholesterol concentration >5.2 mmol/L). Forty-two and 54 patients had a calculated 10-yearr coronary risk of >30% and 20-30%, respectively (risk calculated with the use of the PROCAM algorithm;^17^ 142 patients had metabolic syndrome by National Cholesterol Education Program Adult Treatment Panel III criteria (ATP III).^18^ Patients with a history of established CAD included 9 with unstable angina, 15 with previous myocardial infarction, 10 with a history of coronary artery bypass graft, and 13 with a history of angioplasty. Fifteen patients had undergone a coronary artery bypass graft after a myocardial infarction, and 5 had undergone angioplasty after myocardial infarction (Table 1). The average of lipid profiles of the patients and 10-year coronary risk factor based on Framingham Study before and after 1 year treatment in lipid clinic is shown in Table 2. The lipid profiles of patients attending the lipid clinic over a period of one year improved overall significantly. Serum triglycerides, total cholesterol and LDL-C were reduced by 27.04%, 20.48% and 22.67%, respectively (P<0.001). HDL-C increased by 8.96% (P<0.001). The overall 10-year calculated coronary risk factor of all patients decreased significantly (39.29%, P<0.001, Table 2).

**Table 1:** Characteristics and medication use of dyslipidemic patients.

Patients	n=238
Age	55.2 ± 0.86
Male/Female	143/95
Obese, BMI >30 [*n*(%)]	82 (35)
Type 2 diabetes, fasting blood glucose >7 mmol/L [*n* (%)]	42 (18)
Duration of treatment for diabetes (mo)	13 (0–51)
Established coronary heart disease [*n* (%)]	55 (23)
Unstable angina	9 (4)
MI	15 (6)
CABG	10 (4)
Angioplasty	13 (6)
Angioplasty or CABG after MI	8 (3)
Hypertension [*n* (%)]	186 (79)
High blood pressure (SBP ≥160 mm Hg or DBP ≥100 mm Hg)	76 (32)
Duration of treatment for hypertension (mo)	7.5 (0–72)
Moderate blood pressure (SBP 130-160 mm Hg or DBP 85–100 mm Hg)	110 (46)
Hypertriglyceridemia, serum triglycerides >1.8 mmol/L [*n* (%)]	176 (74)
Hypercholesterolemia, serum total cholesterol >5.2 mmol/L [*n* (%)]	216 (92)
Duration of statin therapy (mo)	9.0 (0–43)
Calculated 10-y coronary risk >30%^1^	42 (18)
Calculated 10-y coronary risk between 20% and 30%	54 (23)
Metabolic syndrome^2^	142 (60)

SBP, systolic blood pressure; DBP, diastolic blood pressure; MI, myocardial infarction; CABG, coronary artery bypass graft.

Median, interquartile range for duration of treatment in parentheses (all such values)

1 Calculated with the PROCAM algorithm.^17^

2 Defined according to National Cholesterol Education Program Adult Treatment Panel III criteria.^18^

**Table 2:** Biochemical characterization of dyslipidemic patients.

n=238	Before treatment	After treatment	Percentage of changes
Triglyceride (mmol/L)	2.7 ± 0.4	1.97 ± 0.17	27.04***
Total cholesterol (mmol/L)	7.47 ± 18	5.94 ± 11	20.48***
LDL-C (mmol/L)	4.72 ± 0.19	3.65 ± 0.10	22.67***
HDL-C (mmol/L)	1.22 ± 0.03	1.34 ± 0.03	8.96***
Ten-year coronary risk factor based on FRAMING-HAM Study	10.18 ± 58.0	6.18 ± 38.0	29.39***

Values are expressed as mean ± SEM.

Comparisons before and after treatment were assessed by paired t-test.

*** P<0.001 for all lipid profiles; LDL-C, low density lipoprotein cholesterol; HDL-C, high density lipoprotein cholesterol

## DISCUSSION

There was a high frequency of obesity, type 2 diabetes, hypertension, and positive smoking habit among the patients that is typical of a lipid clinic population. Our results showed that after one year follow up of the patients in the lipid clinic setting, the status of lipid profiles improved significantly which is consistent with previous investigations within CRRC.^11^–^15^ Pearson et al. (2008) showed that a CRRC can improve lipid levels and suggested that these benefits are sustained once patients are returned to the care of their primary physician.^19^ Our findings confirm the benefits of managing a patient with dyslipidaemia within a lipid clinic setting which can effectively monitor the cardiovascular risk factors of susceptible patients. Several previous randomized trials have shown that the reduction of traditional risk factors especially hypertension and high cholesterol results in a significant reduction in the incidence and recurrence of cardiovascular events.^20^–^23^ There may be several reasons for these findings. First, regular monitoring may ensure compliance with drug therapy. Second, lipid specialists may be more adept at explaining the benefits of treatment and alerting the patients about potential side-effects of medications. General physicians are not likely to be as effective as lipid specialists in this regard.^23^ Our findings highlighted the potential benefits of establishing a specialized lipid clinic for managing dyslipidemic patients. Dyslipidemic patients are often managed by internists or cardiologists in busy non-specialist clinics within the Western and Eastern health systems. Statins are an important medical intervention used within lipid clinics. As is evident from previous studies, they have a pivotal role in lipid lowering of the dyslipidemic patients.^8^ However, there is still controversy concerning target lipid levels, risk, dose adjustment and the most effective systems of patient management and monitoring.^18^

## CONCLUSIONS

The monitoring and management of patients with dyslipidaemia are best undertaken by specialists with a focused clinic. This results in a better response to therapy and attainment of targets for prevention of cardiovascular disease.
